# Small Molecule Inhibitor of Protein Kinase C DeltaI (PKCδI) Decreases Inflammatory Pathways and Gene Expression and Improves Metabolic Function in Diet-Induced Obese Mouse Model

**DOI:** 10.3390/biology13110943

**Published:** 2024-11-18

**Authors:** Brenna Osborne, Rekha S. Patel, Meredith Krause-Hauch, Ashley Lui, Gitanjali Vidyarthi, Niketa A. Patel

**Affiliations:** 1 Research Service, James A. Haley Veteran’s Hospital, 13000 Bruce B Downs Blvd, Tampa, FL 33612, USA; brenna.osborne@va.gov (B.O.); rekha.patel1@va.gov (R.S.P.); meredith.krause-hauch@va.gov (M.K.-H.); gitanjali.vidyarthi@va.gov (G.V.); 2 Department of Molecular Medicine, University of South Florida, Tampa, FL 33612, USA; 3 Department of Molecular Oncology, Moffitt Cancer Center, Tampa, FL 33612, USA; ashley.lui@moffitt.org

**Keywords:** PKCδI, catalytic fragment, adipose tissue, DIO mice, inflammation, TNFα, IL-1β, IL-6, PI3K, AKT, Gas5, Neat1, Malat1, Tmem189, Meg3

## Abstract

Obesity promotes metabolic diseases such as type 2 diabetes and cardiovascular disease. PKCδI is a serine/threonine kinase which regulates cell growth, differentiation, and survival. Caspase-3 cleavage of PKCδI releases the C-terminal catalytic fragment (PKCδI_C), which promotes inflammation and apoptosis. We previously demonstrated an increase in PKCδI_C in human obese adipose tissue (AT) and adipocytes. Subsequently, we designed a small molecule drug called NP627 and demonstrated that NP627 specifically inhibited the release of PKCδI_C in vitro. Here, we evaluate the in vivo safety and efficacy of NP627 in a diet-induced obese (DIO) mouse model. The results demonstrate that NP627 treatment in DIO mice increased glucose uptake and inhibited the cleavage of PKCδI_C in the AT as well as in the kidney, spleen, and liver. Next, RNAseq analysis was performed on the AT from the NP627-treated DIO mice. The results show increases in ADIPOQ and CIDEC, upregulation of AMPK, PI3K-AKT, and insulin signaling pathways, while inflammatory pathways were decreased post-NP627 administration. Further, levels of lncRNAs associated with metabolic pathways were affected by NP627 treatment. In conclusion, the study demonstrates that NP627, a small-molecule inhibitor of PKCδI activity, is not toxic and that it improves the metabolic function of DIO mice in vivo.

## Simple Summary

Obesity results from an excessive level of fat depots and is a global epidemic. Obesity promotes other chronic diseases, such as type 2 diabetes and cardiovascular disease. Adipose (fat cells) is an important endocrine regulator of energy and glucose homeostasis. Protein Kinase Cδ1 (PKCδI) is present in fat cells and regulates their growth and survival. Caspase-3, activated by cell-death signals, cleaves PKCδI and releases a catalytic fragment (PKCδI_C). The PKCδI_C fragment induces inflammation and cell death in adipocytes, advancing the detrimental metabolic effects of obesity. Our prior study had developed a therapeutic, referred to as NP627, to inhibit the release of the PKCδI_C fragment. Previously, the safety, efficacy, and specificity of NP627 have been established using cellular assays with obese adult human adipose stem cells (hASC). The goal of this project was to test the safety and efficacy of NP627 in a diet-induced obese (DIO) mouse model which mimics human obesity. Our results show that NP627 effectively inhibited the release of PKCδI_C, decreased inflammation, and restored glucose metabolism in DIO mice. Unbiased transcriptomics analysis revealed genes that were changed, post-NP627 treatment, in DIO mice. Thus, we believe NP627 has remarkable therapeutic potential for improving the metabolic consequences of obesity.

## 1. Introduction

Obesity is a global epidemic, and its prevalence continues to rise worldwide. The World Health Organization (WHO) reports that in 2022, one in eight people in the world live with obesity [1]. Obesity is known to promote several chronic diseases such as cardiovascular diseases, liver diseases, type 2 diabetes, and cancer. Adipose tissue, an endocrine organ, stores triacylglycerol and is involved in lipogenesis (fatty acid and triglyceride synthesis) and lipolysis (hydrolysis of triacylglycerols) [2]. In addition, adipose tissue secretes adipokines which regulate insulin sensitivity, inflammation, and glucose metabolism. Obesity is a result of hyperplasia and the hypertrophy of adipose tissue, and is characterized by a chronic low-grade state of inflammation, with immune cell infiltration. Lipotoxicity and ectopic fat distribution result from increased systemic free-fatty acid (FFA) release [3]. Obesity results in dysregulation of energy homeostasis, insulin sensitivity, glucose uptake, and inflammation.

The Protein Kinase C (PKC) family was first identified in 1977. They are the central mediators of signal transduction pathways that directly phosphorylate substrates at serine/threonine residues to regulate cell proliferation and gene expression. PKCs are composed of a regulatory domain (N-terminal) and a catalytic domain (C-terminal), separated by a hinge region. The eleven mammalian PKC isoforms are divided into groups based on the second messengers that activate them. Conventional or classical isoforms (PKCα, PKCβ-I, PKCβ-II, and PKCγ) are activated by calcium, diacylglycerol (DAG) and phosphatidylserine (PS). Novel isoforms include PKCδ, PKCθ, PKCε, and PKCη, and are primarily regulated by DAG and PS. Atypical isoforms constitute PKCζ and PKCι/λ.Atypical PKC isoforms act completely independent of both calcium and DAG and are activated primarily by phorbol esters [4,5].

PKCδI (also known as constitutively spliced PKCδ) is a serine/threonine kinase regulating cellular growth, differentiation, and apoptosis [4,6]. The 78 kDa structure of PKCδI can be further divided into four conserved regions (C1-C4) and five variable regions (V1-V5). PKCδI is activated by several different mechanisms [6]. Following phosphorylation of PKCδI at the activation loop (Thr505), turn motif (Ser643), and hydrophobic motifs (Ser662), PKCδI is subsequently released into the cytosol [4,5]. Binding to second messengers such as DAG or PS allows PKCδI to translocate to the plasma membrane, undergo a conformational change, and release the pseudosubstrate domain from the substrate-binding site [4,5,6]. Additionally, apoptotic stimuli such as hydrogen peroxide (H_2_O_2_) will cause tyrosine sites on PKCδI to undergo phosphorylation [7]. Proteolytic activation entails the activation of caspase-3 by apoptotic signals and cleavage of the full-length PKCδI, resulting in release of a 40 kDa catalytic fragment (PKCδI_C). The PKCδI_C can translocate into the mitochondria or the nucleus to induce inflammation and apoptosis [8,9]. PKCδI can also feedback upon itself, activating caspase-3 and further promoting proteolytic cleavage of the catalytic fragment [4]. Previous studies from our lab and others have shown the role of PKCδI in promoting apoptosis and inflammation, as well as increased PKCδI activation in obesity [10,11].

Our prior study [12] demonstrated that human adipocytes derived from obese subjects have increased cleavage of PKCδI, resulting in increased PKCδI_C levels concomitant with increased inflammation. Hence, we developed an inhibitor to prevent the release of PKCδI_C and thereby decrease inflammation in obese adipocytes. Using Schrödinger’s software, we screened for organic small molecules that dually recognized PKCδI and the caspase-3 cleavage site. Molecular dynamics (MD) simulations demonstrated that NP627 bound to PKCδI with high affinity and specificity. Surface plasmon resonance (SPR) kinetics demonstrated that NP627 disrupted the binding of caspase-3 to the hinge region of PKCδI by affecting the macromolecular interactions between these proteins. This was verified by in vitro kinase assays that demonstrated that NP627 specifically inhibited the cleavage of PKCδI and did not affect the activity of other PKC family isozymes. Inhibition of the release of PKCδI_C by NP627 treatment attenuated the phosphorylation of myelin basic protein, a known substrate of PKCδI. These assays utilizing lean and obese human adipose stem cells differentiated to adipocytes demonstrated the in vitro safety and specificity of NP627 and the inhibition of PKCδI activity, and showed that NP627 treatment decreased inflammation and cell death while improving mitochondrial metabolism [12].

In this study, we evaluated the safety and efficacy of NP627 to inhibit the cleavage and release of PKCδI_C in vivo in a diet-induced obese (DIO) mouse model. Further, we performed RNAseq analysis on the adipose tissue samples and identified the differentially regulated genes and pathways that were affected by NP627 in the DIO mouse model.

## 2. Materials and Methods

### 2.1. Animals

**Figure 1:**
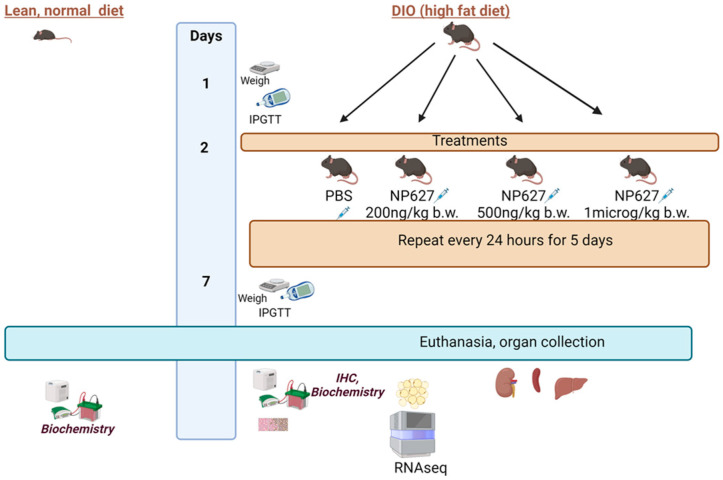
Schematic of study design, created in BioRender. Patel, N (2024). Reprinted with permission from Patel, N (2024). Copyright 2024 Patel, N.

The James A. Haley Veteran’s Hospital and the University of South Florida Institutional Animal Care and Use Committee (IACUC) approved all experimental procedures with animals, consistent with the governing guidelines and recommendations of AWA and HREA. All experiments complied with the ARRIVE guidelines. All mice were raised and studied in pathogen-free environments housed in plastic, sawdust-covered cages with a normal light–dark cycle and free access to chow and water. Ten-week-old male C57BL/6 (lean) and diet-induced obese (DIO) C57BL/6 were purchased from Jackson Laboratories. The mice were acclimated for 2 weeks. DIO mice were continued on a high-fat diet (Research diets, New Brunswick, NJ, USA; Catalog #D12492; 60 kcal% fat) and lean mice were fed a regular diet (Research diets; Catalog #D12450B; 10 kcal% fat). NP627 or PBS (vehicle control) was administered to DIO mice by subcutaneous (SQ) injection; this was repeated daily for 5 days. The NP627 doses administered to DIO mice were 200 ng/kg body weight (b.w.), 500 ng/kg b.w., or 1 μg/kg b.w. of the mice. Mice were euthanized at 13 weeks. The liver, spleen, kidneys, and white adipose tissue from the posterior subcutaneous depot were collected and analyzed from the lean, DIO, and DIO + NP627 mice (n = 5 for each group). The schematic of the study design is shown in Figure 1. The publication license is in Supplemental file S1 (Created in BioRender. Patel, N. (2024) https://BioRender.com/a66o353; accessed on 16 October 2024).

### 2.2. Intraperitoneal Glucose Tolerance Test (IPGTT)

Before treatment began, mice were weighed and fasted for 8 h prior to the initiation of the IPGTT. A single D-glucose solution injection of 2 g/kg was administered. A drop of blood was taken from the tail vein at 0, 30, 60, 90, and 120 min, and these were measured with AlphaTrak blood glucose test strips. An IPGTT was also performed after treatment, and the same protocol was followed. The area under curve (AUC) of blood glucose (BG) was calculated using the following formula: AUC (mmol/L·min) = 1/2 × (BG 0 min + BG 30 min) × 30 min + 1/2 × (BG 30 min + BG 60 min) × 30 min + 1/2 × (BG 60 min + BG 90 min) × 30 min + 1/2 × (BG 90 min + BG 120 min) × 30 min.

### 2.3. Western Blot Analysis

Tissue lysates were prepared by weighing and slicing 1 g of tissue, followed by homogenization using a cell lysis buffer (Cell Signaling, Danvers, MA, USA; Catalog #9803) with 10% protease (Life Technologies, Carlsbad, CA, USA; Catalog #A32953) and phosphatase inhibitors (Life Technologies; Catalog #A32957). A quantum of 70 ug of lysate was loaded on a 10% SDS-PAGE gel and electrophoretically transferred onto a nitrocellulose membrane. The membrane was blocked for one hour with 5% nonfat dried milk in Tris-Buffered saline with 0.05% Tween 20 (TBST) containing 5% bovine serum albumin, and washed before incubation overnight at 4 °C in the primary antibodies. Membranes were probed with antibodies against PKCδI (Santa Cruz Biotechnology, Dallas, TX, USA; Catalog# SC-213; Cell Signaling Technology; Catalog #2058), β-actin (Sigma, St. Louis, MO, USA; Catalog #A3854), GAPDH (GenScript, Piscataway, NJ, USA; Cat #A00191), IL-1β (Cell Signaling; Catalog #12507), and TNFα (Cell Signaling; Catalog #6945). Secondary HRP antibodies were used for rabbit (Bio-Rad, Hercules, CA, USA; Catalog #1706515), goat (ProteinSimple, Santa Clara, CA, USA; Catalog #043-522), and mouse (Invitrogen, Carlsbad, CA, USA; Catalog# 62-6520). Membranes were rocked in chemiluminescent solution (ProteinSimple; Catalog #PS-CS01) for detection of antibodies. Blots were imaged using an Amersham IQ800 (Cytiva, Marlborough, MA, USA) and densitometric units were analyzed using the IQTL Amersham software. All densitometric values were normalized using β-actin as the internal control. Alternatively, automated Western blot was performed using the ProteinSimple Jess system (ProteinSimple) according to the manufacturer instructions. A 1 mg/mL sample was loaded, and antibodies were used at a 1:10 dilution. Each capillary was normalized to total protein loaded and GAPDH, and analyzed based on chemiluminescence signal peaks. Compass Software (ProteinSimple v 6.3.0) was used for automated Western blot analysis. Original Western blots are in Supplemental file S2.

### 2.4. Hematoxylin and Eosin Staining

The kidney, spleen, liver, and adipose tissue were harvested from DIO (PBS) and DIO mice treated with NP627 and sliced 30–35 μm thick; the organs were placed on gelatin coated slides. Staining was performed the day after cryo-sectioning with the Hematoxylin and Eosin (H/E) kit (Abcam, Waltham, MA, USA; Catalog #ab245880) according to the manufacturer’s instructions. H/E stain was added for durations ranging from 30 s to 2 min, depending on the organ. Slides were rinsed in water to remove the excess stain before dehydration with alcohol washes (70%, 95%, and 100% ethanol for 2 min each). Then, Permount mounting medium (Fisher Chemical, Pittsburg, PA, USA; Catalog #SP15-500) was used to mount the cover slide. Slides were imaged using the Keyence BZ-X810 digital integrated microscope.

### 2.5. Quantitative Real-Time qPCR

Total RNA was isolated from adipose tissue samples from DIO and DIO + NP627 mice by use of RNAzol RT (Molecular Research Center Inc., Cincinnati, OH, USA; Catalog #RN190) according to the manufacturer’s instructions. A sample of 1 µg of RNA was used to synthesize cDNA. A 1 μL portion of cDNA was then amplified by real-time quantitative PCR with SYBR Green Master Mix (Applied Biosystems, Carlsbad, CA, USA; Catalog #4472908) using the ABI ViiA7 qPCR machine (Applied Biosystems). The primers used were as follows: IL-1β S 5′-CTCGTGGTGTCGGACCCATATGA-3′, IL-1β AS 5′-TGAGGCCCAAGGCCACAGGT-3′, TNFα S 5′-GGTGCCTATGTCTCAGCCTCTT-3′, TNFα AS 5′-GCCATAGAACTGATGAGAGGGAG-3′, IL-6 S 5′-TCCGGAGAGGAGACTTCACA-3′, IL-6 AS 5′-TGCAAGTGCATCATCGTTGT-3′, β-actin S 5′- TGTCCACCTTCCAGCAGATGT-3′, and β-actin AS 5′- AGCTCAGTAACAGTCCGCCTAGA-3′. RT q-PCR was performed in triplicates on samples and standards. Primer concentrations were optimized to generate a standard curve and a single-melt curve. Lean adipose tissue samples were used as a calibrator, and β-actin was used as the endogenous control to determine the relative quotient, using the ∆∆CT method. A standard curve was generated for every primer used to determine the absolute quantification (AQ). For the standard curve, 1 µL cDNA from the lean sample was used for PCR, and amplification was performed on the Biometra. The concentration of the amplified PCR product was measured using the DeNovix (DS-11). A serial dilution was then performed on the amplified PCR product in the following dilutions: 1:10, 1:100, 1:1000, 1:10,000, and 1:100,000. RT-qPCR was performed on the ABI ViiA7, using the standard curve protocol. The dsDNA concentration of the 1:10 dilution and dilutions thereafter were calculated and entered in the ABI ViiA7. After the RT-qPCR run, expression levels were uploaded to a Microsoft Excel spreadsheet where an AQ equation and standard curve were generated.

### 2.6. RNAseq and Analysis

Adipose tissue samples from lean, DIO, and DIO treated 500 ng/kg b.w. NP627 were homogenized, and total RNA was extracted with the Qiagen RNeasy plus minikit (Qiagen Sciences, Germantown, MD, USA, Catalog #74134). The Qubit and Agilent Tape Station were used for measuring RNA concentration and quality. It was ensured that RIN was >8.0 for all samples. The library was prepared using the TruSeq stranded mRNA Library Prep Kit according to the manufacturer’s instructions (Illumina, San Diego, CA, USA, Cat#: 20040532). The concentration and quality of the resulting DNA library was checked using the Qubit and Agilent Tape Station. Samples were loaded into the Illumina NextSeq 500, with 75 bp pair-end reads with indices. The TruSeq System Suite (Illumina) was utilized for real-time image analysis and base calling. All samples had a minimum of 40 million reads and sequences aligned to >80% to the reference genome. The Trimmomatic (v 0.36) was used to trim reads, and then a quality check was performed using FASTQC. Reads were mapped using HISAT2 (v 2.1.0) to mouse genome GRCm39 (file downloaded from NCBI). The files were converted using SAMtools (v 1.3.1) and Feature-Counts (v 1.6.3) were used to determine reads. RStudio (v 2023.09.0 + 463) was used for analysis of differentially expressed genes (DEGs) and the results were further analyzed using Qiagen’s Ingenuity pathway analysis (IPA) and the Kyoto Encyclopedia of Genes and Genomes (KEGG) to generate a clustered heatmap, distance heatmap, venn diagram, volcano plot, and pathways.

### 2.7. Statistical Analysis

All experiments were repeated three times to verify the reproducibility of results. Statistical analysis was performed using GraphPad PRISM™ software (v 10.0.0). Student’s *t*-test or Welch’s *t*-test, paired parametric, and unpaired *t*-test were used to analyze results for significant changes between groups (* *p* < 0.05, ** *p* < 0.01, *** *p* < 0.001, and **** *p* ≤ 0.0001; ns = not significant).

## 3. Results

### 3.1. NP627 Administration Does Not Affect the Weight of DIO Mice

**Figure 2:**
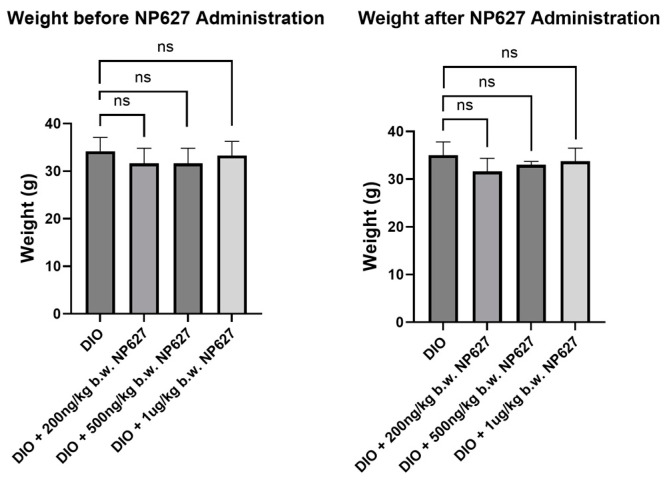
Mice were weighed before and after NP627 administration, and their respective weights were graphed. Statistical analysis using Student’s *t*-test was performed; ns = not significant.

In this study, we sought to evaluate the safety and efficacy of NP627 in a diet-induced obese (DIO) mouse model. DIO mice were weighed before and after NP627 treatments to determine whether NP627 affected their weight. The DIO mice were treated with subcutaneous (SQ) injections of NP627 (200 ng/kg b.w., 500 ng/kg b.w., or 1 μg/kg b.w.) or PBS (vehicle control: DIO) in the abdomen, performed daily for 5 days. The results show no significant changes in body weight in DIO mice post-NP627 treatments (Figure 2), indicating that NP627 treatment does not result in weight loss in DIO mice.

### 3.2. IPGTT Shows Increased Glucose Uptake with NP627 Treatment in DIO Mice

**Figure 3:**
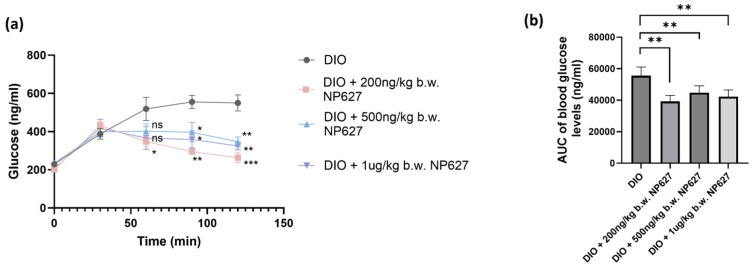
(**a**) An IPGTT was performed. Glucose readings were taken every 30 min for 2 h. Statistical analysis was performed using Welch’s *t*-test, * *p* < 0.05; ** *p* < 0.01; *** *p* ≤ 0.001; ns = not significant. (**b**) The area under the curve (AUC) was calculated as AUC (mmol/L·min) = 1/2 × (BG 0 min + BG 30 min) × 30 min + 1/2 × (BG 30 min + BG 60 min) × 30 min + 1/2 × (BG 60 min + BG 90 min) × 30 min + 1/2 × (BG 90 min + BG 120 min) × 30 min. BG = blood glucose. Data are expressed as mean ± SEM, n = 5. Statistical analysis was performed using a paired parametric *t*-test, ** *p* < 0.01.

DIO mice were treated with a subcutaneous (SQ) injection of NP627 or PBS (vehicle control) in the abdomen, performed daily for 5 days, as described above. We evaluated glucose uptake post-NP627 treatment using the Intraperitoneal Glucose Tolerance Test (IPGTT), which measures the clearance of glucose from the body. The IPGTT was performed before and after NP627 treatment. The results (Figure 3a,b) showed DIO mice had decreased glucose clearance, and that administration of NP627 to DIO mice increased glucose uptake. These results show that NP627 improved the glucose metabolism in DIO mice.

### 3.3. NP627 Inhibited the Cleavage of PKCδI Catalytic Fragment in DIO Mice

**Figure 4:**
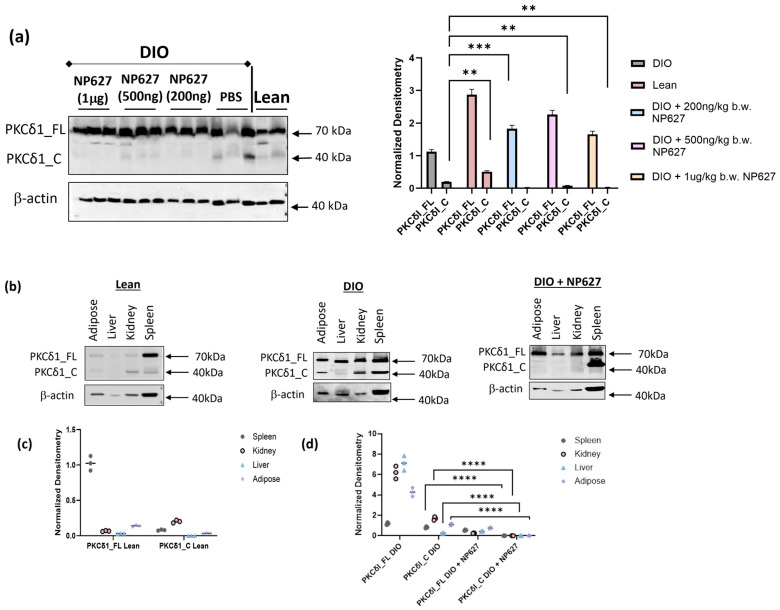
(**a**) Total protein was extracted from adipose tissue of lean, DIO, and DIO mice treated with NP627 (200 ng/kg b.w., 500 ng/kg b.w., or 1 μg/kg b.w.), and a Western blot was performed using antibodies against PKCδI and β-actin. Samples were run in triplicate. Densitometric units obtained by Amersham IQTL analysis software were normalized to β-actin. Welch’s *t*-test was used to determine statistical significance: ** *p* < 0.01; *** *p* ≤ 0.001. (**b**) Total protein levels from the adipose tissue, liver, kidney, and spleen from lean, DIO, and DIO + 200 ng/kg b.w. NP627 mice were analyzed by Western blot, and a representative blot is shown. (**c**) Graph of densitometric units of 3 lean mice were calculated by Amersham IQTL software and normalized to β-actin. (**d**) Graph of densitometric units of 3 DIO mice and 3 DIO + 1 μg/kg b.w. NP627 mice were calculated by Amersham IQTL software and normalized to β-actin. Statistical analysis was performed in GraphPad using Welch’s *t*-test, **** *p* ≤ 0.0001, comparing DIO versus DIO + 1 μg/kg b.w. NP627.

Previously, we demonstrated that adipocytes from obese subjects had increased cleavage of PKCδI by caspase-3 compared to adipocytes from lean subjects, and that the therapeutic NP627 inhibited the release of the catalytic fragment in vitro in obese adipocytes. Hence, we evaluated the adipose tissue (AT) from lean, DIO (PBS, vehicle control), and DIO mice treated with NP627 (200 ng/kg b.w., 500 ng/kg b.w., or 1 μg/kg b.w.). Western blot analysis was performed using an antibody against PKCδI that recognizes both the PKCδI full length protein (PKCδI_FL) and the catalytic fragment (PKCδI_C). The results show an increase in the PKCδI_C in DIO mice compared to lean mice (Figure 4a), which is in concurrence with results from the human obese adipose tissue. The results show that one mouse from DIO (PBS) cohort had low protein loading, as determined by the corresponding β-actin levels. Treatment with 200 ng/kg b.w., 500 ng/kg b.w., or 1 μg/kg b.w. NP627 inhibited the release of PKCδI_C in DIO mice. The graph represents densitometric units normalized to corresponding β-actin levels grouped by treatment cohorts. The results demonstrate that release of PKCδI_C was inhibited by administration of NP627 in DIO mice.

NP627 was administered by SQ injection. From prior research [13,14], it is known that drugs injected via SQ can enter the circulation and affect other organs. Hence, we evaluated additional organs for the efficacy of NP627. Adipose tissue as well as the liver, kidney, and spleen from lean, DIO (PBS, vehicle control), and DIO mice treated with 1 μg/kg b.w. NP627 were evaluated by Western blot analysis. The results (Figure 4b) demonstrate that PKCδI_C was cleaved in the adipose tissue, kidney, and spleen in DIO mice, but not in the lean mice. However, the liver did not exhibit high levels of PKCδI_C in DIO mice. Treatment of DIO mice with NP627 inhibited the release of PKCδI_C in the organs. Equal protein was loaded in each lane, and the results show varying β-actin levels in the organs. The graph (Figure 4c) shows the densitometric units from lean mice normalized to the respective β-actin levels. The graph in Figure 4d shows the statistical analysis of densitometric units in DIO versus DIO+NP627 mice. These results demonstrate that SQ injection resulted in a systemic distribution of NP627 and lowered the PKCδI_C levels in other organs simultaneously with its lowering of PKCδI_C in the adipose tissue.

### 3.4. NP627 Treatment Is Not Toxic

**Figure 5:**
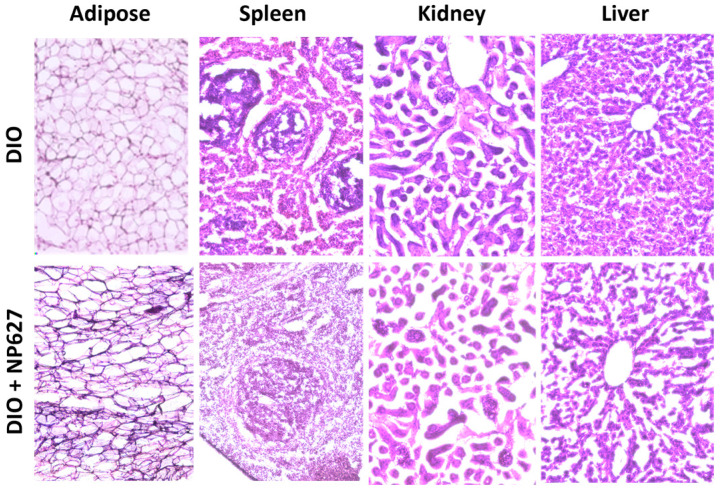
Hematoxylin and eosin staining was performed on the adipose tissue, kidney, spleen, and liver on DIO (PBS) and DIO mice treated with 1 μg/kg b.w. NP627. Slides were imaged using Keyence BZ-X810.

Hematoxylin and eosin staining was performed on the adipose, spleen, kidney, and liver of DIO mice treated with PBS (vehicle) or NP627 1 μg/kg b.w. (highest dose). The results (Figure 5) demonstrate that the NP627 treatment was not toxic to any organ.

### 3.5. RNAseq Analysis Identifies Pathway Changes in Response to NP627 Treatment

**Figure 6:**
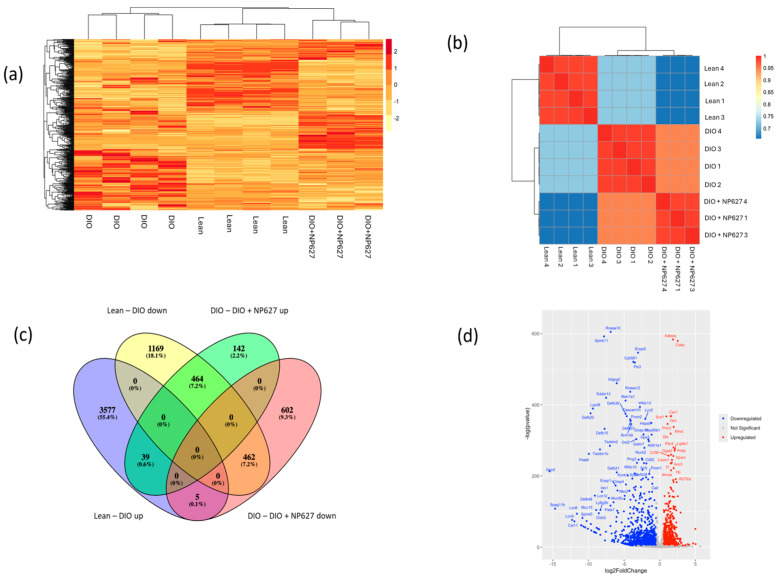
(**a**) Clustered heatmap of differentially expressed genes (DEGs) between lean, DIO, and DIO + NP627. (**b**) Distance heatmap showing how closely related the samples are within their respective groups. (**c**) Venn diagram demonstrating upregulated and downregulated genes that were significantly reversed due to NP627 treatment. (**d**) Volcano plot of genes that differ significantly between lean, DIO, and DIO + NP627 groups.

To investigate the changes in gene expression and pathways, RNAseq analysis was performed on adipose tissue from DIO (PBS, vehicle) mice and DIO mice treated with NP627 (500 ng/kg b.w.). The raw RNAseq data is in Supplemental Table S1. The distance heatmap analysis cluster of groups indicates that treatment with NP627 reversed gene expression in DIO, making it closer to lean expression (Figure 6a). The distance heatmap shows correlation between each sample (Figure 6b). A Venn plot was generated for analysis of up- or downregulated genes after administration of NP627 (Figure 6c). There were 1169 genes that were downregulated in DIO, compared to the lean. Administration of NP627 to DIO resulted in upregulation of 464 genes. The results also show that five genes that were upregulated in DIO compared to lean were significantly downregulated by NP627 administration. The volcano plot (Figure 6d) indicates Adipoq and Cidec to be the most significantly upregulated genes post-NP627 treatment, while Rnase 10 and Spink11 were significantly downregulated post-NP627 treatment.

**Figure 7:**
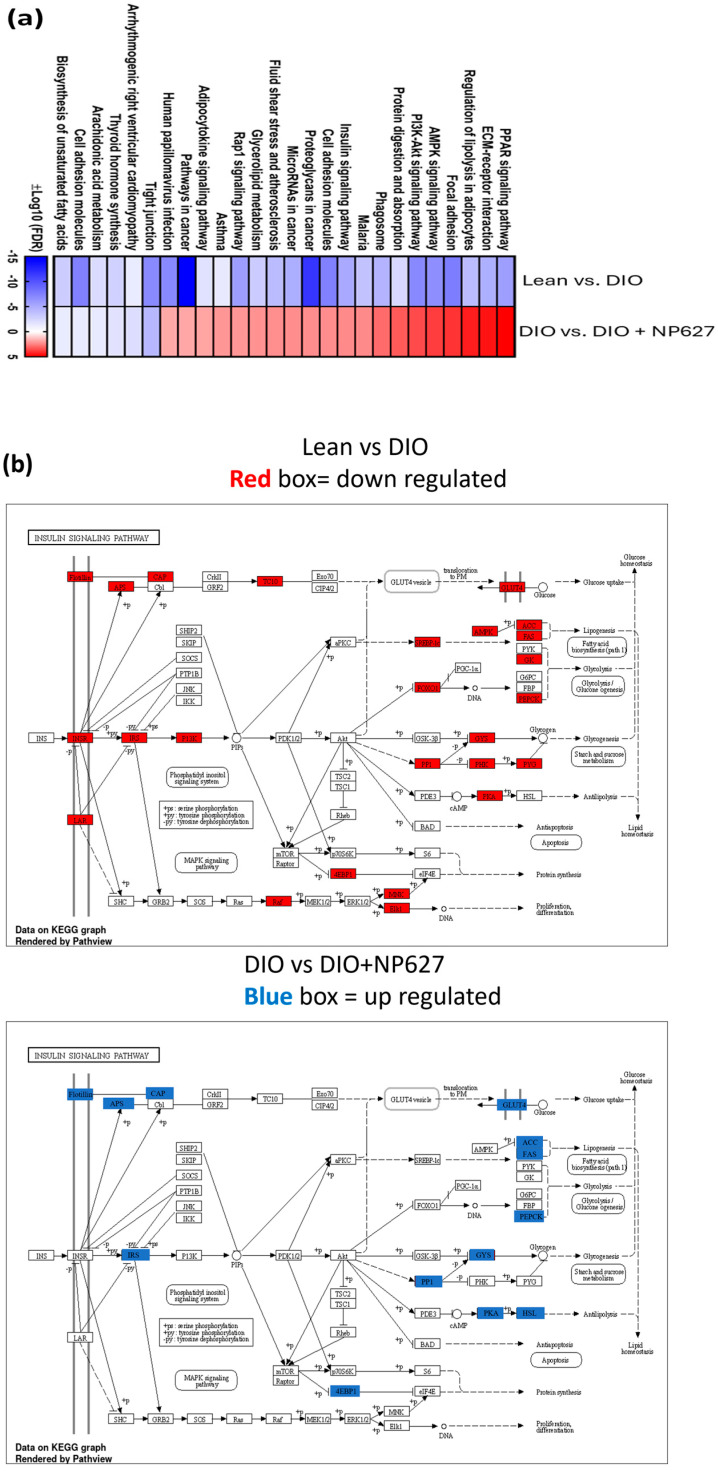

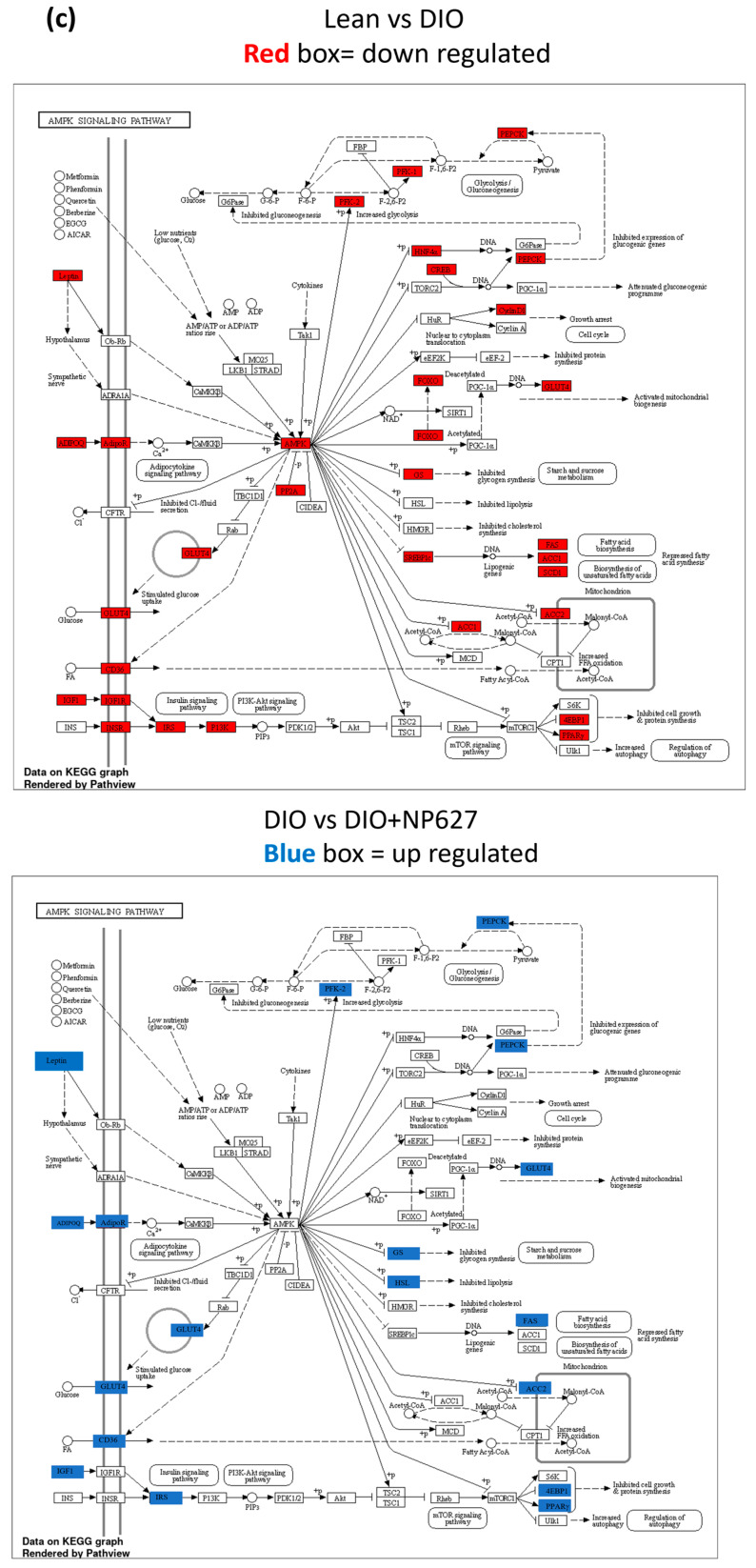

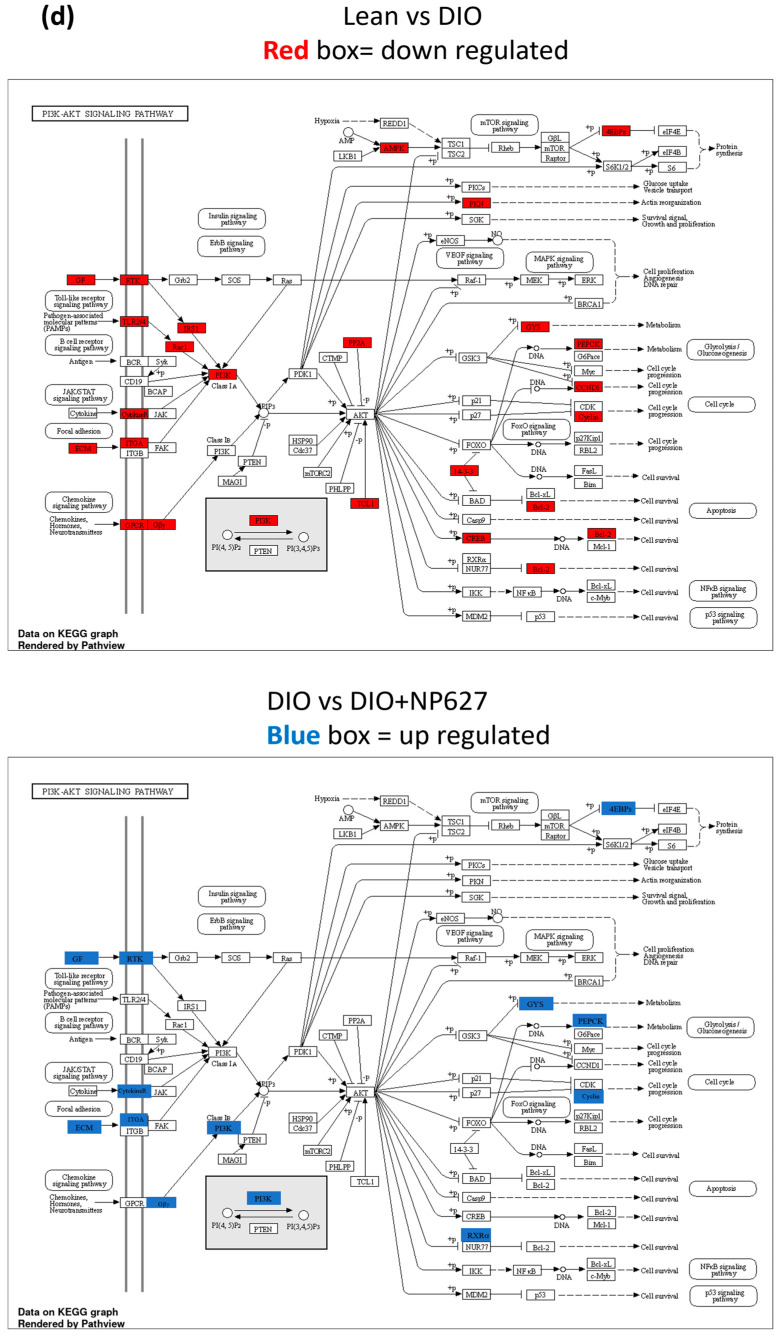

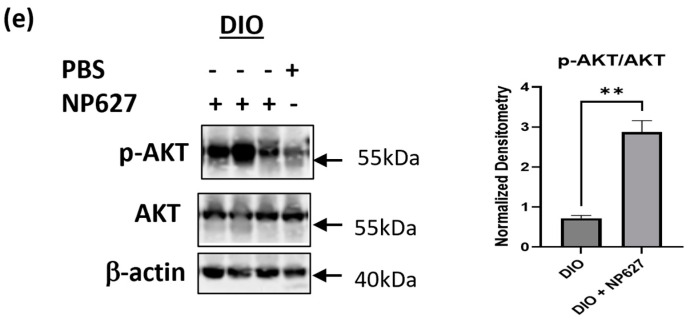
(**a**) A pathway enrichment table was created showing pathways that were upregulated/downregulated between their respective groups. Blue indicates significantly downregulated pathways, and red indicates significantly upregulated pathways. Blue represents a smaller log10 false discovery rate (FDR), representing a more significant correlation. Red represents a larger log10 FDR. (**b**) KEGG pathway mapping of the insulin-signaling pathway. (**c**) KEGG pathway map of the AMPK-signaling pathway. (**d**) KEGG pathway map outlining genes of the PI3K-AKT-signaling pathway. (**e**) Western blotting using p-AKT, AKT, and β-actin antibodies was performed on adipose tissue from DIO and DIO + NP627 mice. β-actin was used for normalization purposes. Densitometric units from the immunoblot were calculated using Amersham IQTL analysis software. Statistical significance was determined in GraphPad using a parametric paired *t*-test, ** *p* < 0.01.

Pathway enrichment analysis using KEGG was performed to investigate changes in DIO vs. DIO + NP627 mice. Enriched pathways (Figure 7a) that were significantly activated in response to NP627 treatment included the insulin-signaling pathway, AMPK pathway, and PI3K-AKT-signaling pathway. Using KEGG, these pathways were visualized. The genes that were downregulated in lean versus DIO mice are shown in red boxes, while the genes that were upregulated in DIO mice post-NP627 treatment are shown in blue boxes in the insulin-signaling pathway (Figure 7b), AMPK pathway (Figure 7c), and the PI3K-AKT-signaling pathway (Figure 7d). These results show that the genes that were affected by NP627 treatment in DIO mice were integral to metabolic signaling pathways.

To verify the analysis, we evaluated the phosphorylation of AKT, which is regulated by all of these pathways (i.e., the insulin-signaling pathway, AMPK pathway, and PI3K-AKT-signaling pathway). Western blot analysis indicated that NP627 treatment in DIO mice increased phosphorylation of AKT (Figure 7e). The results demonstrate that NP627 treatment increases activation of metabolic pathways in DIO mice.

### 3.6. NP627 Reduced Inflammatory Genes TNFα, IL-6, and IL-1β

**Figure 8:**
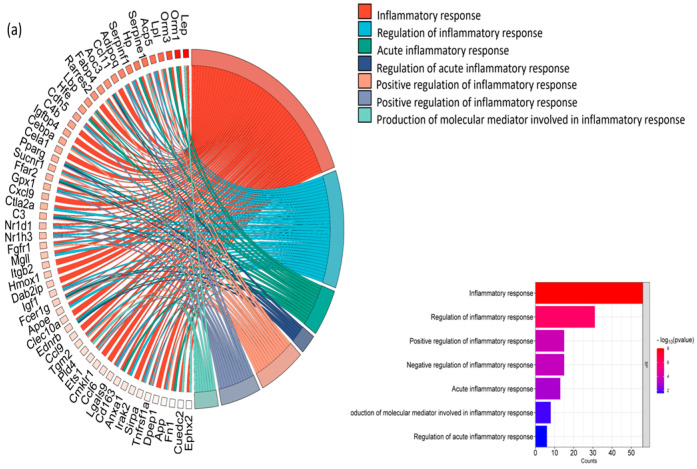

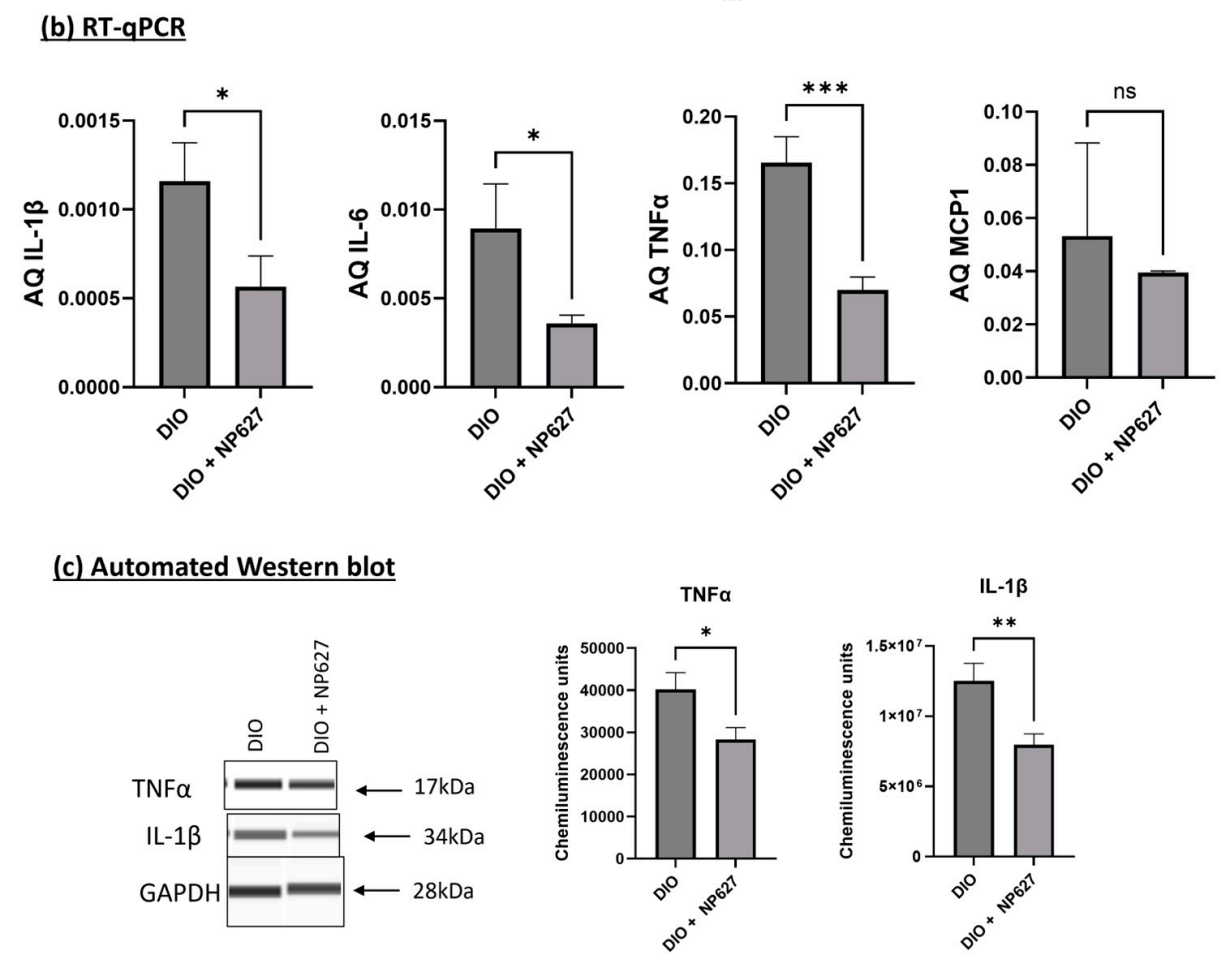
(**a**) Chord diagram showing relationship between genes and inflammatory pathway nodes. (**b**) Total RNA was extracted from adipose tissue of DIO and DIO + NP627 mice. Real-time qPCR was performed in triplicate using SYBR Green to measure the absolute quantification (AQ) of inflammatory genes IL-1β, IL-6, TNFα, and MCP-1, and normalized with β-actin. Statistical analysis was performed in GraphPad using an unpaired *t*-test: * *p* < 0.05, *** *p* < 0.001, and ns = not significant. (**c**) Western blot analysis was performed on adipose tissue from DIO, and DIO + NP627 mice using JESS (automated Western blotting), using antibodies against TNFα and IL-1β. The graph represents ± SEM chemiluminescence units calculated by JESS software Compass. Statistical analysis was performed in GraphPad using Welch’s *t*-test: * *p* < 0.05, ** *p* < 0.01.

Our previous publication demonstrated that PKCδI_C cleavage and release increased inflammation in vitro. Hence, we evaluated the responses to NP627 of the genes from the inflammatory pathways by using the chord diagram, which shows the connections between the genes and pathway nodes (Figure 8a). Next, we validated individual genes in the inflammation pathways using RT-qPCR (Figure 8b) and automated Western blotting (ProteinSimple JESS, Figure 8c). The results demonstrate that TNFα, IL-6, and IL-1β expression were decreased by NP627 treatment in DIO mice. MCP1 levels did not change significantly in response to NP627 treatment. These results demonstrate that NP627 treatment decreased inflammation in DIO mice.

### 3.7. NP627 Treatment Affects Expression of lncRNAs

**Figure 9:**
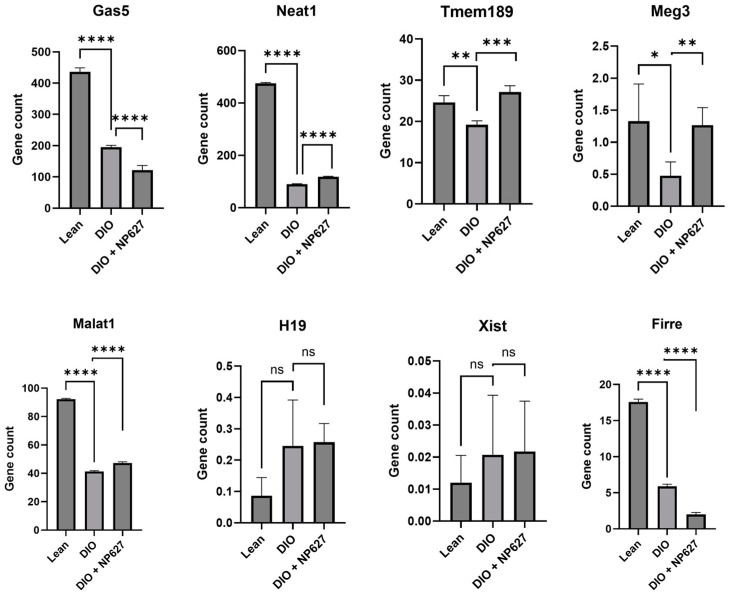
RNAseq data was analyzed for lncRNA expression in lean vs DIO vs DIO + NP627. Graph represents FKPM (fragments per kilobase of exon per million fragments mapped) gene counts (n = 4). Statistical significance was determined in GraphPad using Welch’s *t*-test; * *p* < 0.05, ** *p* < 0.01, *** *p* < 0.001, **** *p* ≤ 0.0001, ns = not significant.

Long noncoding RNA (lncRNA) are regulators of gene expression, and aberrant expression is implicated in multiple diseases, including obesity. We analyzed the RNAseq data for lncRNAs that were changed in DIO mice compared to lean mice and then evaluated the effect of NP627 on the expression of the lncRNAs. The consistently detected lncRNAs across all mice cohorts included Gas5, Neat1, Tmem189, Meg3, Xist, Malat1, H19, and Firre. Amongst these, Gas5, Neat1, Tmem189, Meg3, Malat1, and Firre were decreased in DIO mice compared to lean mice, while H19 and Xist were increased in DIO mice, though the increase was not statistically significant. Treatment with NP627 in DIO mice increased the levels of Neat1, Tmem189, and Meg3 while Gas5 and Firre levels were further decreased with NP627 treatment in DIO mice, compared to DIO mice (Figure 9). Treatment with NP627 did not affect the levels of Xist and H19 in DIO mice. These results indicate that only specific lncRNAs were affected by NP627 treatment in DIO mice. These lncRNAs were previously shown to regulate expression of genes in type 2 diabetes and obesity, a finding which is further described in the Section 4.

## 4. Discussion

Adipose tissue is a critical endocrine regulator of the body’s energy, glucose homeostasis, and inflammation. Obesity is characterized by chronic low-grade inflammation. Chronic metabolically dysfunctional adipose tissue in obesity promotes the development of metabolic syndrome, encompassing insulin resistance, glucose intolerance, dyslipidemia, hypertension, and heart disease [2,15,16]. PKCδI, a diacyl glycerol (DAG)-dependent kinase, is proteolytically activated by cleavage at its hinge region by caspase-3, leading to the release of the catalytic fragment (PKCδI_C). Excessive PKCδI_C leads to increased apoptosis and inflammation [6].

**Figure 10:**
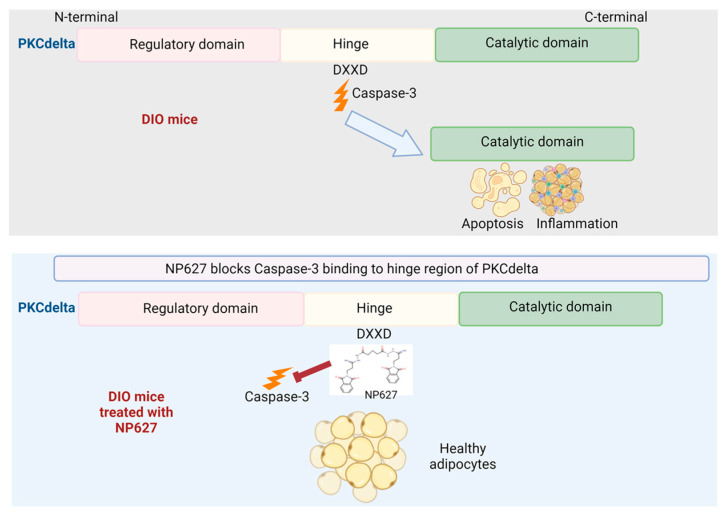
Schematic of action of NP627, created in BioRender. Patel, N (2024). Reprinted with permission from Patel, N (2024). Copyright 2024 Patel, N.

Our prior study [12] demonstrated that PKCδI_C levels were increased in human adipocytes derived from obese individuals concurrent with inflammation. We had demonstrated that PKCδI_C increased inflammation in obese adipocytes in vitro. Then, we developed a small-molecule inhibitor of PKCδI activity called NP627, which was designed to bind to the caspase-3 binding site at the amino acid sequence DXXD(P4-P1)/X on the PKCδI V3 region [12]. NP627 inhibited the binding of caspase-3 to PKCδI and attenuated the release of PKCδI in vitro in human ASC and adipocytes derived from obese subjects. In this study, we evaluated the in vivo efficacy of NP627 in DIO mice. Our results demonstrate that NP627 inhibited the release of PKCδI_C in vivo and was not toxic. We previously demonstrated the specificity of NP627 and showed that it did not inhibit the kinase activity of the other PKC isozymes PKCα, PKCβ, PKCγ, PKCε, PKCθ, or PKCζ, or splice variants of mouse PKCδI, including PKCδII [15]. The results here also demonstrate that the full-length PKCδI_FL levels did not change with administration of NP627. This indicates that the activity of PKCδI_FL in adipocytes, which is required for regulation of reactive oxygen species levels and regulation of lipoprotein lipase, as well as mediating NADPH oxidase activity [11,17,18,19,20], remains intact post-NP627 treatment. In summary, we demonstrate that NP627 administration to DIO mice inhibits the release of PKCδI_C (catalytic fragment), thereby decreasing inflammation and apoptosis and promoting metabolically healthy adipocytes (schematic in Figure 10). The publication license is in Supplemental file S3 (Created in BioRender. Patel, N. (2024) https://BioRender.com/i02z264; accessed on 16 October 2024).

Glucose transporter type 4 (GLUT4) in adipose tissue promotes the uptake and clearance of glucose from blood [21]. We examined the effect of NP627 on glucose metabolism by performing an IPGTT before and after treatment. The IPGTT showed decreased glucose uptake in DIO mice, a condition which was rescued by NP627 treatment. The pathways analysis of RNAseq results showed that GLUT4 levels were increased post-NP627 treatment. Prior research has indicated that upregulation of GLUT4 in adipose tissue improves insulin sensitivity; however, this is, by itself, not enough to protect against the damaging effects of diet-induced glucose intolerance [21]. TNFα is known to suppress the expression of many proteins necessary for modulation of insulin-stimulated glucose uptake in adipose tissue, namely, insulin receptor (IR), insulin receptor substrate-1 (IRS-1), and GLUT4 [22]. Higher expression levels of TNFα in DIO mice might play a part in the latter’s ability to metabolize glucose.

Hyperplasia of adipocytes in obesity causes hypoxia, insulin resistance, and increased caspase-activation, leading to inflammation and apoptosis [23,24]. Obesity is accompanied by low-grade chronic inflammation in adipocytes. Here, the results demonstrate an increase in inflammatory genes in DIO mice. In lean populations, macrophages alternatively are activated (M2) and secrete anti-inflammatory cytokines. In contrast to this, an obese state causes M2 macrophages to polarize and switch to classically activated M1 macrophages. M1 macrophages secrete pro-inflammatory cytokines including TNFα, IL-6, and IL-1β [24,25,26]. TNFα is notably increased in an obese state, and the secretion of TNFα comes primarily from the M1 macrophage [25,27]. Further, TNFα is critical for regulation of insulin signaling locally in adipose tissue as well as for controlling lipogenesis and lipolysis [27]. It should be noted that insulin resistance is often the first noticeable defect in type 2 diabetes disease progression [27]. It has been proposed that a deficiency of TNFα in high-fat-diet-fed mice protects against insulin resistance and improves glucose metabolism [2]. Caspase-8 and Caspase-3 were also seen to be significantly increased with the presence of TNFα [28]. Our results here demonstrated higher expression levels of TNFα in DIO mice, which were subsequently reduced by NP627 treatment. IL-1β has been shown to increase lipolysis [25]. Elevated serum glucose and FFAs β-cells in the pancreas release IL-1β [25]. With NP627 treatment, IL-1β expression levels were significantly decreased in DIO mice. Research has shown that high levels of IL-1β downregulate IRS1, GLUT4, PI3K, and pAKT [25]. IL-1β is also known to act on PPARγ to inhibit adipogenesis [25].

KEGG pathway analysis of RNAseq results showed that NP627 affected genes in the AMPK-signaling, PI3K-AKT-signaling, and insulin-signaling pathways, which are implicated in obesity [29,30,31]. Furthermore, dysfunctional PI3K-AKT signaling is known to promote obesity [29]. Insulin resistance further exacerbates PI3K-AKT signaling [29]. Current diabetic medications target the PI3K-AKT-signaling pathway [29]. In the RNAseq volcano plot, genes that were upregulated included Adipoq and cell death-inducing DNA fragmentation factor-like effector C (Cidec). Adipoq, encoding adiponectin, regulates energy and glucose homeostasis, along with lipid metabolism, and research has determined that adiponectin levels are lowered in an obese state [32]. Low adiponectin levels promote the development of type 2 diabetes, cardiovascular disease, and insulin resistance [32]. Current research focusing on obesity targets Adipoq for therapeutic intervention [32]. Cidec expression is localized to adipose tissue, and it plays many imperative roles in fat metabolism [33]. The cidec gene is directly associated with insulin sensitivity in humans [33]. Interestingly, RNase 10 and Spink11 were seen to be downregulated. RNase 10 plays a vital role in sperm maturation, motility, and fertilization [34]. Spink11 also plays a part in male fertility [35]. Some of the adipose tissue harvested was close to the epididymal fat pad explaining the expression of male fertility genes. Ectonucleotide pyrophosphatase/phosphodiesterase family member 5 (ENPP5) was found to be downregulated. ENPP5 is a biomarker for the onset of insulin resistance [36]. According to the GO analysis, NP627 was seen to upregulate lipid transport and downregulate stem cell population maintenance and Wnt signaling. Wnt signaling acts through several pathways to regulate basic cellular functions such as proliferation, differentiation, cell cycle progression, and determination of cells’ fates [37].

The levels of the lncRNAs Gas5, Neat1, Tmem189, Meg3, Xist, Malat1, H19, and Firre were decreased in DIO mice. NP627 treatment in DIO mice affected these levels, indicating that activity of PKCδI_C may be directly or indirectly regulating the transcription of these lncRNAs. Future studies will evaluate the underlying mechanisms mediated by PKCδI_C and used to affect levels of these lncRNAs. Gas5 has been shown to be reduced in type 2 diabetes [38,39]. Meg3 levels have previously been shown to be low in obesity [40], and our results concur with the earlier finding that showed the role of Meg3 in glucose homeostasis and insulin signaling. Our results in adipose tissue indicate that inhibition of release of PKCδI_C further reduces Gas5, while increasing the levels of Meg3 in DIO mice, suggesting a PKCδI-dependent pathway promoting the onset of type 2 diabetes in obesity. The lncRNAs Neat1 and Malat1 were reduced in DIO mice, a finding in concurrence with previous results [41,42,43,44]. Neat1 is shown to regulate PPARγ-splicing in adipocytes [45], while Malat1 is involved in fat deposition as well as adipogenesis [42]. H19 has been shown to be involved in epigenetic regulation of adipocyte differentiation [46]. Xist regulates differentiation of brown adipocytes via C/EBPα [47]. Tmem189 is also implicated in brown adipocyte differentiation [48]. The role of Firre is not yet described in adipocytes, to the best of our knowledge, though a recent publication implicated Firre in modulating inflammation in macrophages [48]. In this study, the RNAseq did not include small ncRNAs; we will undertake an scRNAseq in the future for the purpose of a global transcriptomics analysis.

The limitations of this study are that it was conducted using only male C57BL/6 mice, and that the drug NP627 was administered for 5 days, reflecting an acute response. Future studies will compare the trends for female C57BL/6 mice and a longer duration of NP627 treatment will be studied. The IPGTT showed that 200 ng/kg b.w. performed better than the 1 μg/kg b.w. dose. To understand this finding, a separate study is presently evaluating the pharmacodynamics and pharmacokinetics of NP627. Here, we have demonstrated the efficacy of NP627 in vivo to inhibit the release of PKCδI_C and reduce inflammation in a DIO mouse model. We have also established the effect of NP627 on pathways critical in insulin resistance and obesity.

## 5. Conclusions

The study established NP627 to be an inhibitor of PKCδI_C and demonstrated its efficacy in vivo in a DIO mouse model. Overall, the results show that NP627 promotes a metabolically healthy adipose tissue by decreasing inflammation. Several novel targets of PKCδI_C were identified, which will be pursued in future studies. Overall, NP627 is a specific small-molecule therapeutic which mitigates detrimental effects of PKCδI_C in obesity.

## 6. Patents

Patent awarded to N.A.P. for PKCδI inhibitor NP627: US Patent No. 11,844,779B2 PKCdeltaI (PKCδI) inhibitor formulations and uses thereof with utilities (19 December 2023).

## Data Availability

The raw data of the RNAseq are uploaded as supplemental data.

## Supplementary Materials

The following supporting information can be downloaded at: 916 https://www.mdpi.com/article/10.3390/biology13110943/s1, Table S1: The raw RNAseq FPKM data. File S1: BioRender Publication Permission for schematic of study design. File S2: The original Western blots. File S3: BioRender Publication Permission for schematic of NP627 action.
