# Supplementary figures and images for: Small Molecule Inhibitor of Protein Kinase C DeltaI (PKCδI) Decreases Inflammatory Pathways and Gene Expression and Improves Metabolic Function in Diet-Induced Obese Mouse Model
Source: Biology (Basel). 2024 Nov 18;13(11):943. doi: 10.3390/biology13110943 (PMC11591907; doi:10.3390/biology13110943)

Figure 4a

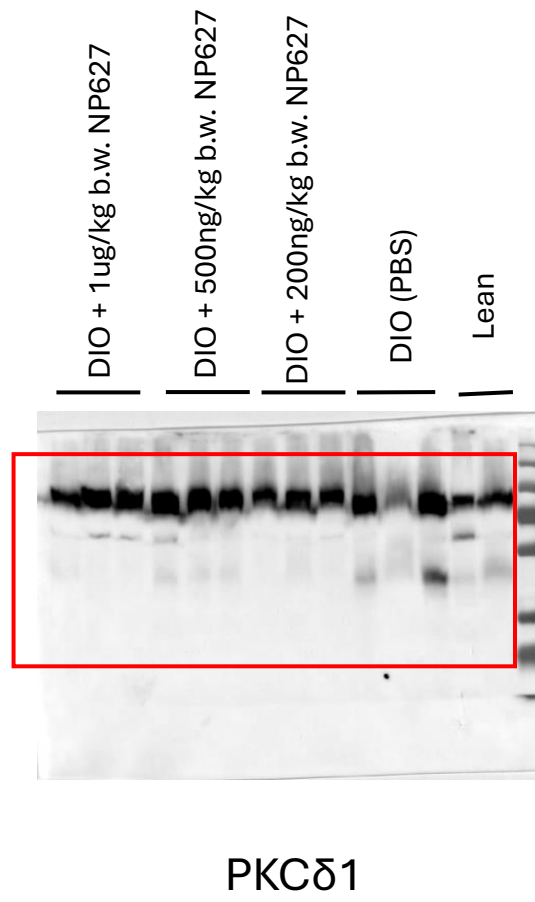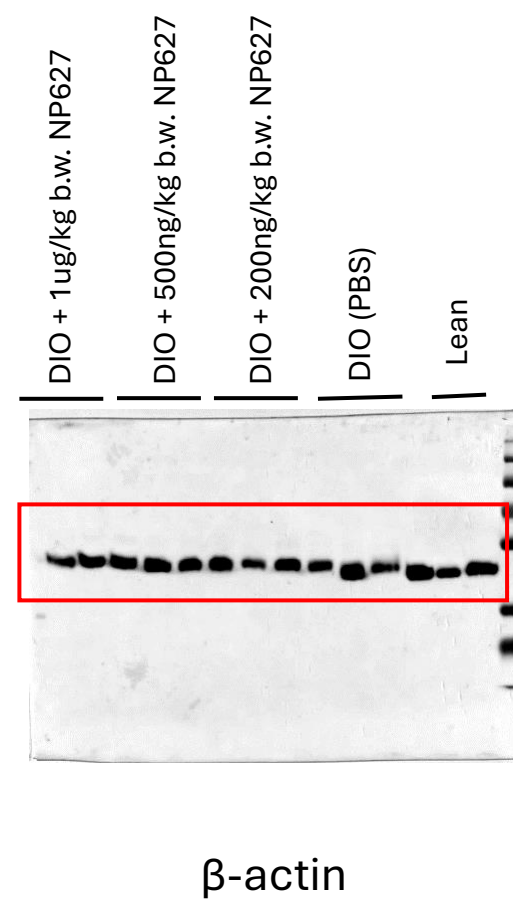

Figure 4b

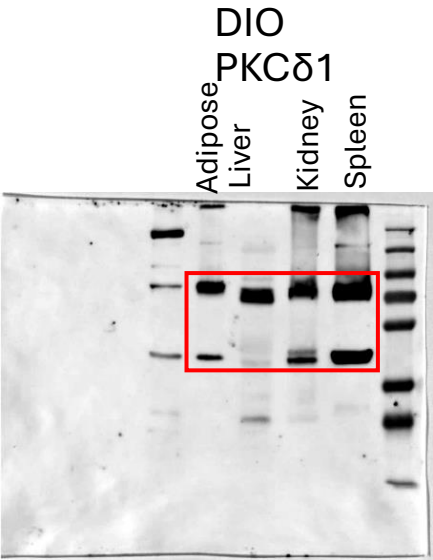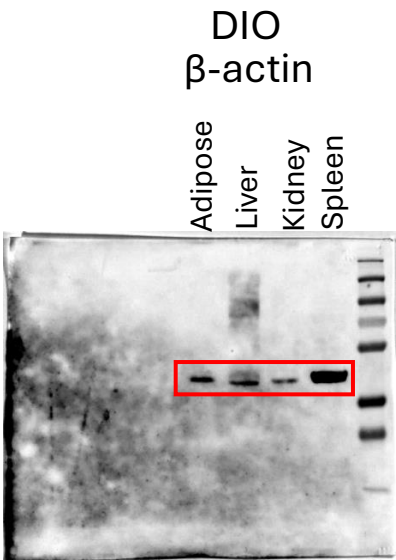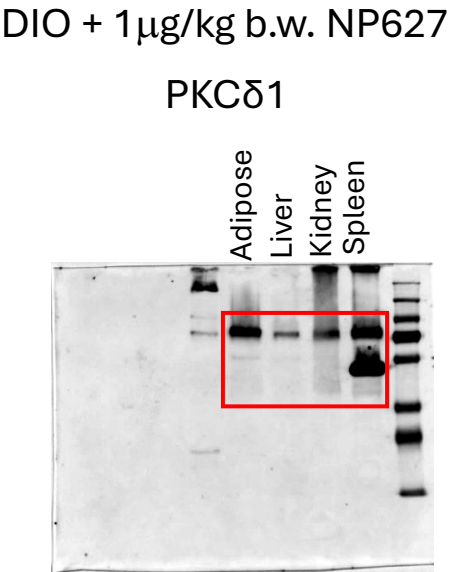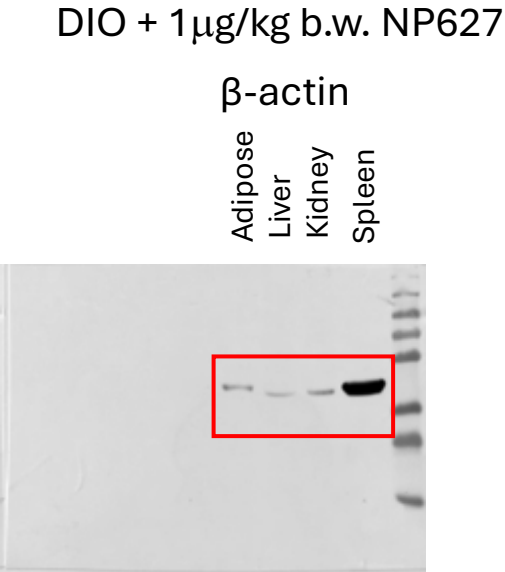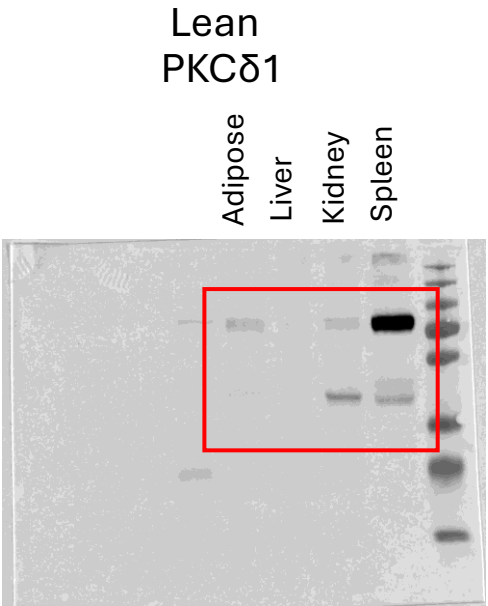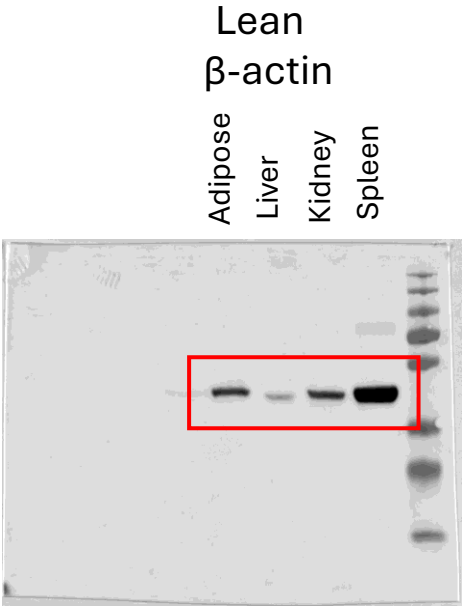

Figure 6e

AKT

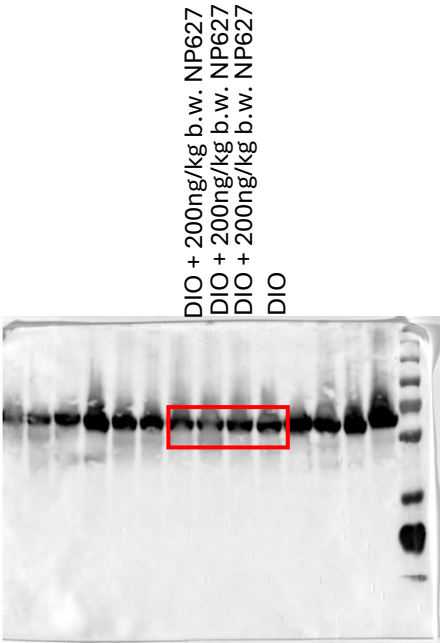

p-AKT

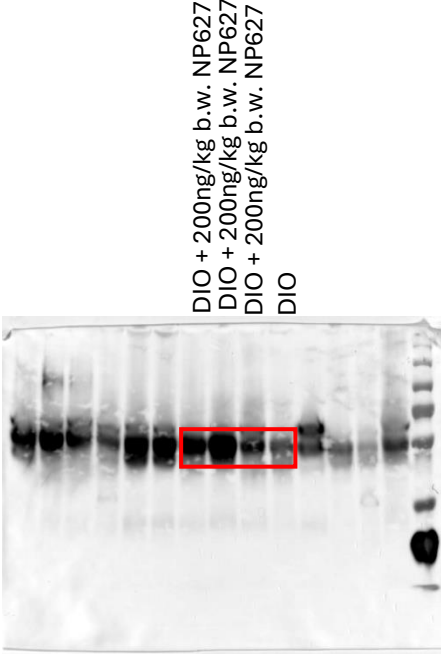

$\beta$ -actin

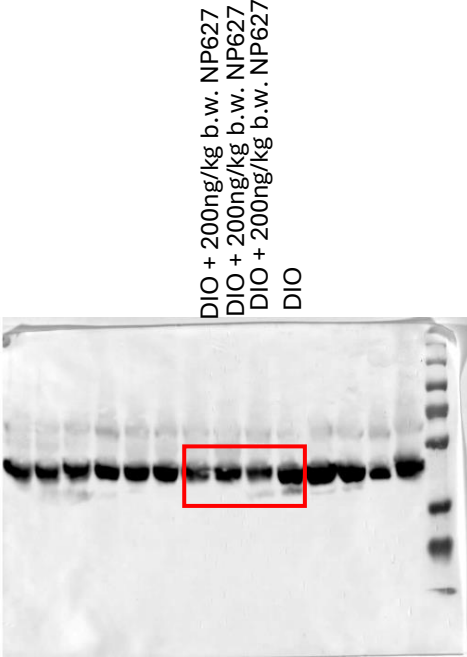

Figure 8c

TNF $\alpha$

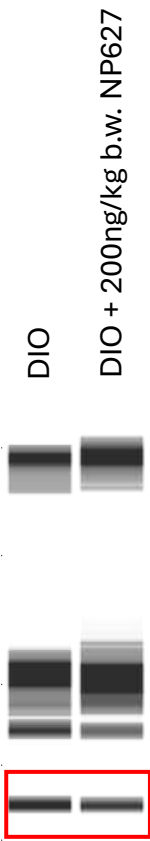

IL-1 $\beta$

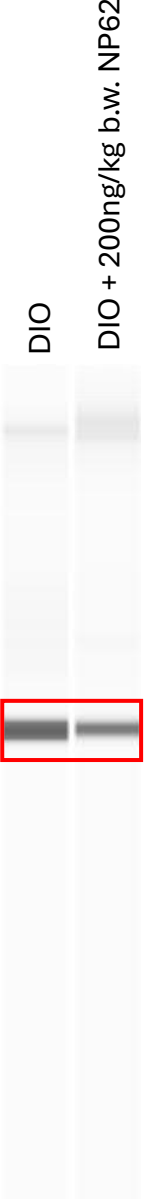

GAPDH

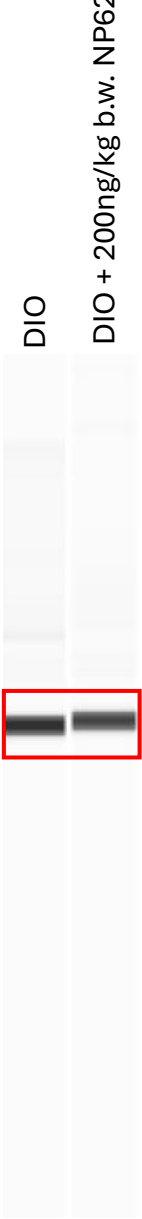

Supplement: Supplementary file 1 [file biology-13-00943-s001.zip › Supple S2 NP627 DIO original blots.pdf]
